# Substitutions of Zr^4+^/V^5+^ for Y^3+^/Mo^6+^ in Y_2_Mo_3_O_12_ for Less Hygroscopicity and Low Thermal Expansion Properties

**DOI:** 10.3390/ma12233945

**Published:** 2019-11-28

**Authors:** Qiang Ma, Lulu Chen, Heng Qi, Qi Xu, Baohe Yuan, Xiansheng Liu, Lei Xu

**Affiliations:** 1 Collage of Electric Power, North China University of Water Resources and Electric Power, Zhengzhou 450011, China; mqiang1977@ncwu.edu.cn (Q.M.); chenlulu@ncwu.edu.cn (L.C.); hqi1292@163.com (H.Q.); hnxuq@ncwu.edu.cn (Q.X.); yuanbaohe@ncwu.edu.cn (B.Y.); 2 Henan Key Laboratory of Photovoltaic Materials and Low Dimensional Materials Science Laboratory, Henan University, Kaifeng 475004, China

**Keywords:** hygroscopicity, thermal expansion, substitutions, microstructure

## Abstract

In this investigation, Zr*_x_*Y_2−*x*_V*_x_*Mo_3−*x*_O_12_ (0 ≤ *x* ≤ 1.4) is developed and the effects of the substitutions of Zr^4+^/V^5+^ for Y^3+^/Mo^6+^ in Y_2_Mo_3_O_12_ on the hygroscopicity and thermal expansion property are investigated. For the smaller substitution content (*x* ≤ 0.5), their crystal structures remain orthorhombic, while there is crystal water still in the lattice. The linear coefficients of thermal expansions (CTEs), for *x* = 0.1, 0.3, 0.5, and 0.7, are about −4.30 × 10^−6^, −0.97 × 10^−6^, 0.85 × 10^−6^, and 0.77 × 10^−6^ K^−1^, respectively, from 476 to 773 K, which means that the linear CTE could be changed linearly with the substitution content of Zr^4+^/V^5+^ for Y^3+^/Mo^6+^ in Y_2_Mo_3_O_12_. As long as the substitution content reaches *x* = 1.3/1.4, almost no hygroscopicity and low thermal expansion from room temperature are obtained and are discussed in relation to the crystal structure and microstructure.

## 1. Introduction

Most materials expand when heated, which come from anharmonic lattice vibrations, which increases the distance between atoms. There are abnormal materials, which contract when heated and are called negative thermal expansion (NTE) materials. NTE materials have attracted tremendous attention due to their potential applications in manufacturing zero thermal expansion devices and matching the coefficients of thermal expansion (CTEs) between different materials [1,2,3]. The mechanisms of NTE are extensively investigated [4,5,6,7]. More and more NTE materials have being discovered [8,9,10,11,12,13]. The NTE properties come from different mechanisms for different crystal structures, such as the magnetostrictive effect [14,15], anharmonic lattice vibration effect [16], ferroelectric polarization effect [1], and charge transfer effect [17]. There are many experimental means to research NTE mechanisms: Neutron/X-ray pair distribution function analysis, extended X-ray absorption fine structure, infrared absorption spectrum, Raman scattering spectroscopy, etc. All of these gradually promote the practical applications of NTE materials.

However, some drawbacks of NTE materials limit their practical application, such as narrow NTE temperature range, smaller NTE coefficient, metastable structure, hygroscopicity, much higher phase transition temperature than room temperature (RT), etc. [18,19,20,21]. A_2_M_3_O_12_ family materials have wide NTE temperature ranges and stable structures. However, the A^3+^ ion radius can affect their phase transition temperature and hygroscopicity. For example, Fe_2_Mo_3_O_12_ crystallizes in monoclinic structures at RT and a phase transition from monoclinic to orthorhombic structure on takes place at high temperatures and then NTE could present. Y_2_Mo_3_O_12_ has heavy hygroscopicity in the atmospheric environment at RT, with the crystal water limiting the transverse vibration of bridge oxygen atom in A-O-M linkage (it is responsible for NTE). NTE could only be present after releasing crystal water. The hygroscopicity of Y_2_Mo_3_O_12_ could be reduced by using the ion-substitution (Ce^3+^ [22], Fe^3+^ [23], La^3+^ [24], and (LiMg)^3+^ [25]) method. Aliovalent substitution of Mo^6+^ and W^6+^ for V^5+^ in ZrV_2_O_7_ does not change its structure [26,27]. Dual-ion substitutions could change the phase temperature and thermal expansion coefficient [8,9,10,28]. Dual-ion substitutions of Fe^3+^/Mo^6+^ for Zr^4+^/V^5+^ in ZrV_2_O_7_ can obtain near zero thermal expansion material [8].

In order to avoid hygroscopicity of Y_2_Mo_3_O_12_ and to obtain controllable thermal expansion coefficient material, partial substitution may be necessary for both Y and Mo sites and may require enhancing the solubility of dopants. In this work, we develop Y_2−*x*_Zr*_x_*Mo_3−*x*_V*_x_*O_12_ material using Zr^4+^/V^5+^ to substitute Y^3+^/Mo^6+^ in Y_2_Mo_3_O_12_, and investigate the substitution effects on their thermal expansion properties and hygroscopicity in detail. It is shown that the linear CTE could be changed linearly with the substitution content of Zr^4+^/V^5+^ for Y^3+^/Mo^6+^ in Y_2_Mo_3_O_12_. A near zero thermal expansion is obtained when *x* = 1.3/1.4.

## 2. Materials and Methods

Y_2−*x*_Zr*_x_*Mo_3−*x*_V*_x_*O_12_ (0.0 ≤ *x* ≤ 1.4) were synthesized by a solid-state method using Y_2_O_3_, ZrO_2_, MoO_3_, and V_2_O_5_ as raw materials. The raw materials were mixed according to stoichiometric amounts of Y_2_O_3_, ZrO_2_, MoO_3_, and 3% excess V_2_O_5_ of desirable materials and were then ground in a mortar for 3 h and pressed into tablets with measuring about 10 mm in length and diameter 10 mm. This was followed by sintering at 1073 K (0.0 ≤ *x* ≤ 0.7) and 973 K (*x* = 1.3/1.4) for 4 h and cooling naturally to RT.

The linear thermal expansion coefficients of Y_2−*x*_Zr*_x_*Mo_3−*x*_V*_x_*O_12_ were measured on a dilatometer (LINSEIS DIL L76, Linseis Messgeräte GmbH, Selb, Germany). Thermogravimetric analysis (TG) of the powder samples were performed with an Ulvac Sinku-Riko DSC (Model 1500M/L, ULVAC, Methuen, MA, USA) in the temperature range of 298–873 K with the heating and cooling rates of 10 K/min. XRD measurements of the powder samples were carried out with an X-ray diffractometer (Model X’Pert PRO, Malvern Panalytical Ltd., Malvern, UK) to identify the crystalline phase. Raman spectroscopy (Renishaw MR-2000 Raman spectrometer, Renishaw, Wotton-under-Edge, UK) was used to characterize the vibrational property of lattice. The microstructures of the as-prepared pristine and modified block Y_2_Mo_3_O_12_ were recorded using a scanning electron microscopy (SEM, FEI Quanta 250, FEI, Hillsboro, OR, USA) with an accessory of energy dispersive spectrometer (EDS).

## 3. Results and Discussion

### 3.1. Low Thermal Expansion and Hygroscopicity

**Figure 1:**
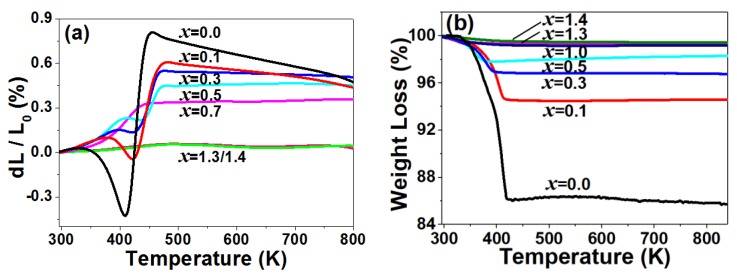
(**a**) Relative length change and (**b**) thermogravimetric analysis (TG) of Y_2−*x*_Zr*_x_*Mo_3−*x*_V*_x_*O_12_.

Figure 1a shows the relative linear length change curves of Y_2−*x*_Zr*_x_*Mo_3−*x*_V*_x_*O_12_ (0.0 ≤ *x* ≤ 1.4). For the sample with *x* = 0.1 in Y_2−*x*_Zr*_x_*Mo_3−*x*_V*_x_*O_12_, the thermal expansion property was similar to the reported Y_2_Mo_3_O_12_ (*x* = 0.0) [22,23], indicating that less substitution of Zr/V for Y/Mo in Y_2_Mo_3_O_12_ could not change the crystal structure of Y_2_Mo_3_O_12_. However, for *x* = 0.3, the thermal contraction and thermal expansion corresponding to the release of adsorbed and crystal water before 476 K reduced remarkably, indicating obvious reduction of the hygroscopicity (Figure 1b) [29]. The NTE trend after the release of crystal water (>476 K) lowered, suggesting that the translation of bridge O in Y-O-Mo and the rotation of polyhedraYO_6_/MoO_4_ related to NTE were limited by the substitutions of Zr/V for Y/Mo in Y_2_Mo_3_O_12_. For the weight loss curve of the sample *x* = 0.5, after the release of crystal water, there was a slight weight increase, which should have been related to instrument error.

The detailed estimation of the linear CTEs of the samples with *x* = 0.0, 0.1, 0.3, 0.5, and 0.7 were about −8.18, −4.30 × 10^−6^, −0.97 × 10^−6^, 0.85 × 10^−6^, and 0.77 × 10^−6^ K^−1^, respectively, from 476 to 773 K, suggesting that the linear CTE could be changed gradually with the substitution amount of Zr^4+^/V^5+^ for Y^3+^/Mo^6+^ in Y_2_Mo_3_O_12_. Additionally, even near zero thermal expansion could be realized for *x* = 0.7. It is interesting that, so long as the substitution reached *x* = 1.3/1.4, RT was observed almost without hygroscopicity and low thermal expansion (Figure 1a,b).

### 3.2. Crystal Structure

**Figure 2:**
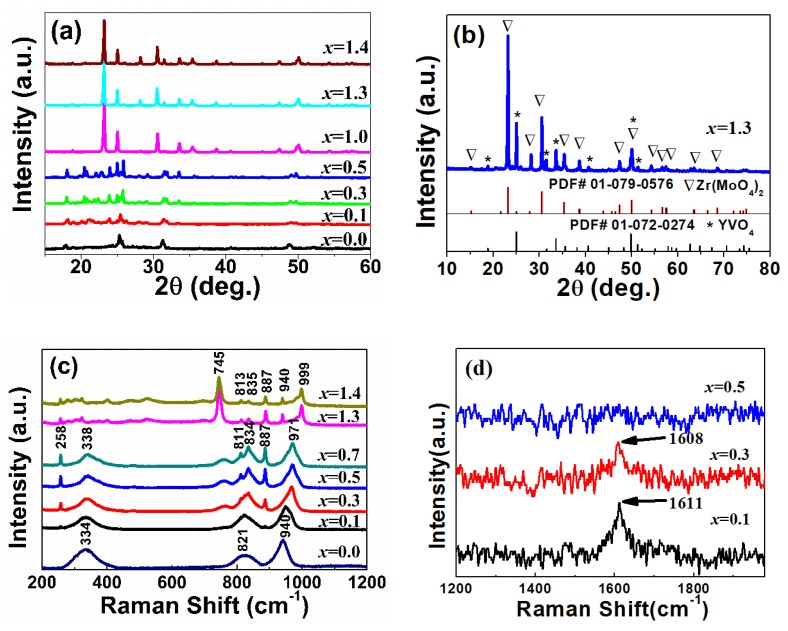
XRD patterns (**a**,**b**) and Raman spectra (**c**,**d**) of Y_2−*x*_Zr*_x_*Mo_3−*x*_V*_x_*O_12_.

To make the relationship between the thermal expansion property and crystal structure change clear, with the substitution amount of Zr^4+^/V^5+^ for Y^3+^/Mo^6+^ in Y_2_Mo_3_O_12_, XRD patterns and Raman spectra of Y_2−*x*_Zr*_x_*Mo_3−*x*_V*_x_*O_12_ were performed. Figure 2a shows the XRD patterns of Y_2−*x*_Zr*_x_*Mo_3−*x*_V*_x_*O_12_ at RT with *x* = 0.0, 0.1, 0.3, 0.5, 1.0, 1.3, and 1.4 (for comparison, the XRD patterns of the samples with *x* = 0.2, 0.4, and 0.7 are also presented in Figure S1). For *x* = 0.0, there were only four diffraction peaks corresponding to the fully hydrated form of Y_2_Mo_3_O_12_·3H_2_O [22,23,24,25]. The XRD spectra had minor changes with the increase of Zr^4+^/V^5+^, without diffraction peaks from other materials (such as ZrMo_2_O_8_, YVO_4_, ZrO_2_, and ZrV_2_O_7_), which agreed with the results of the thermal expansion. These mean that there was crystal water in the lattice. For *x* = 0.3–0.5, the diffraction peaks corresponded to that of Y_2_Mo_3_O_12_, with the space group of Pbcn. However, for *x* ≥ 1.0, the primary diffraction peaks corresponded to Zr(MoO_4_)_2_ or YVO_4_ (Figure 2b), which indicated that there was a mixture of Zr(MoO_4_)_2_ and YVO_4_.

Figure 2c shows the Raman spectra of Y_2−*x*_Zr*_x_*Mo_3−*x*_V*_x_*O_12_. For *x* = 0.1, there were three primary Raman bonds similar to 940, 821, and 334 cm^−1^ and that of Y_2_Mo_3_O_12_, due to the presence of crystal water [22,23,25]. Additionally, two weak peaks at 258 and 887 cm^−1^ were also detected, which should have been related to Zr-O or V-O vibrations. In particular, for *x* = 0.7, the three bonds became sharp, presented a blue shift, and even split to two peaks (821 cm^−1^ → 811 and 834 cm^−1^) [23], which suggested the decreased effect of crystal water. For *x* = 1.3/1.4, the Raman peaks changed so much that the main peak was replaced by the peak at 745 cm^−1^, which should have been related to the formation of new phases corresponding to the result from XRD patterns (Figure 2a).

Figure 2d shows a high wave number Raman spectra of Y_2−*x*_Zr*_x_*Mo_3−*x*_V*_x_*O_12_. There was only one bond at about 1611 cm^−1^ for *x* = 0.1 and 0.3, which was ascribed to the bending (υ_2_ + υ_4_) vibrations of OH^−^. This indicated that there was crystal water in the samples. However, this bond disappeared for *x* ≥ 0.5, which meant that the substitutions of Zr/V for Y/Mo could inhibit the entry of water into the lattice.

**Figure 3:**
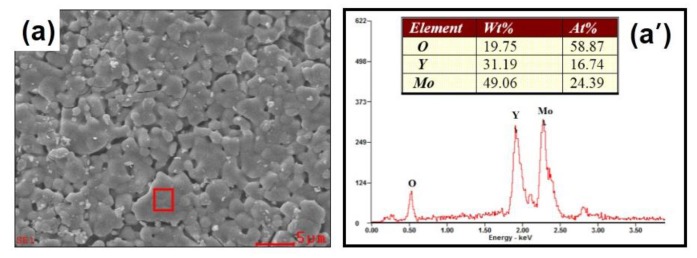

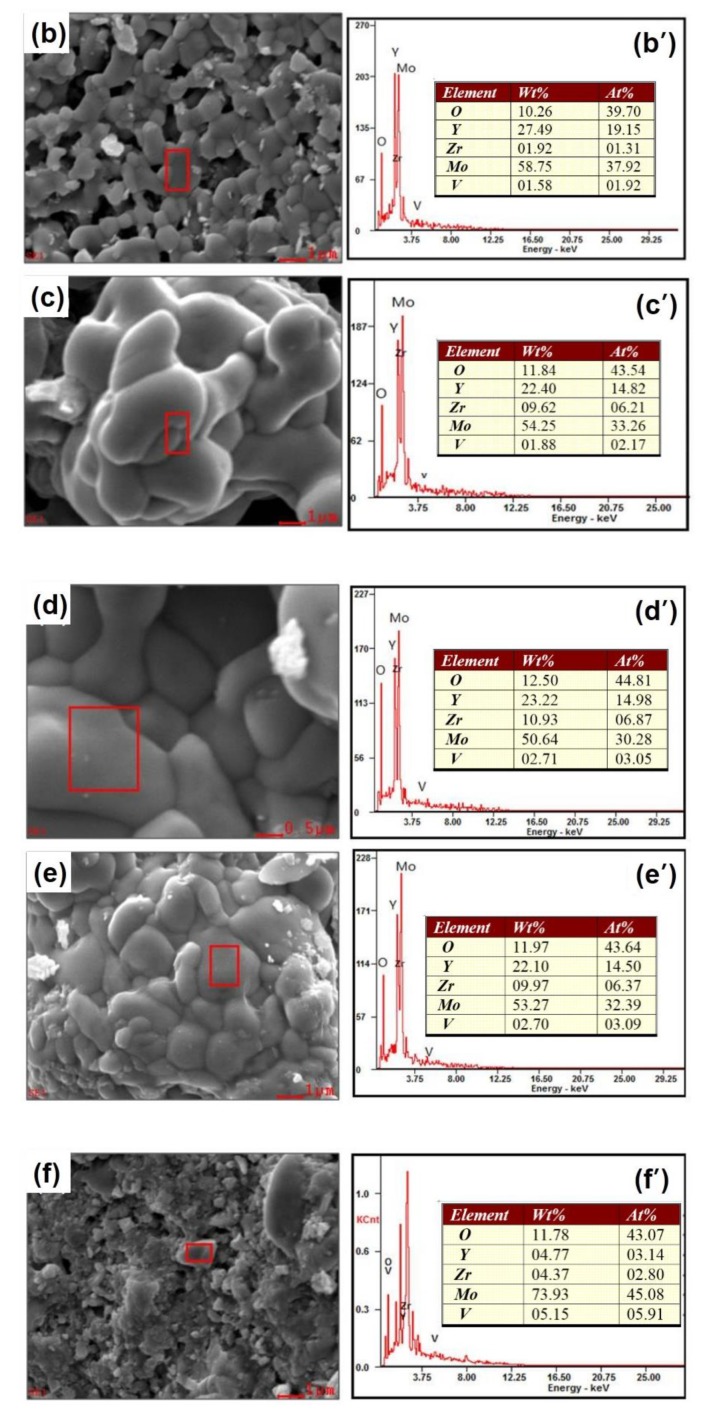

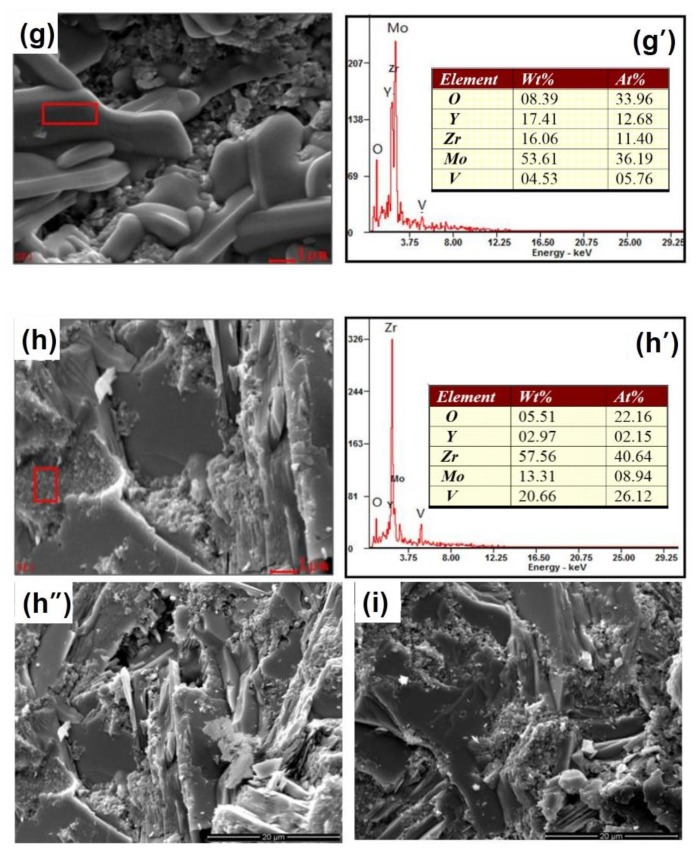
Scanning electron microscopy (SEM) images (**a**–**h**) and the corresponding energy dispersive spectrometer (EDS) spectra (**a**′–**h**′) of Y_2−*x*_Zr*_x_*Mo_3−*x*_V*_x_*O_12_ for *x* = 0.0, 0.1, 0.2, 0.3, 0.4, 0.5, 0.7, and 1.4, respectively, and SEM images (**h**″ and **i**) of *x* = 1.4 and 1.3.

In order to further check the effect of the substitution of Zr^4+^/V^5+^ for Y^3+^/Mo^6+^ on the hygroscopicity of Y_2_Mo_3_O_12_, the microstructures of Y_2−*x*_Zr*_x_*Mo_3−*x*_V*_x_*O_12_ were performed by SEM and EDS spectra (Figure 3a–g and Figure 3a′–g′ for *x* = 0.0, 0.1, 0.2, 0.3, 0.4, 0.5, 0.7, and 1.4, respectively). For *x* = 0.1, the SEM image showed aggregation grains separated from some pores due to the heavy hygroscopicity (Figure 3a) [22,29]. For *x* = 0.2 and 0.3, the aggregation grains were bigger than that of 0.1, and the number of pores become less and less due to the reduction of hygroscopicity (Figure 3b,c). For *x* = 0.4, grains bonded together just like the binder, and far less pores were observed (Figure 3d). This means that the hygroscopicity of Y_2_Mo_3_O_12_ was suppressed by the substitution of Zr^4+^/V^5+^ for Y^3+^/Mo^6+^. However, amorphous grains were observed for *x* = 0.5 (Figure 3e), and even for *x* = 0.7, there were several, big, long columnar particles with smooth surfaces relating to the grain melting (Figure 3f). The EDS spectrum of *x* = 0.7 presented elements of Zr, V, Y, Mo, and O (Figure 3f′). For *x* = 1.3 and 1.4, the small grains and laminar structures were observed, which could relate to the two phases of Zr(MoO_4_)_2_ and YVO_4_, which corresponded to the XRD result (Figure 3g,g″,h).

**Figure 4:**
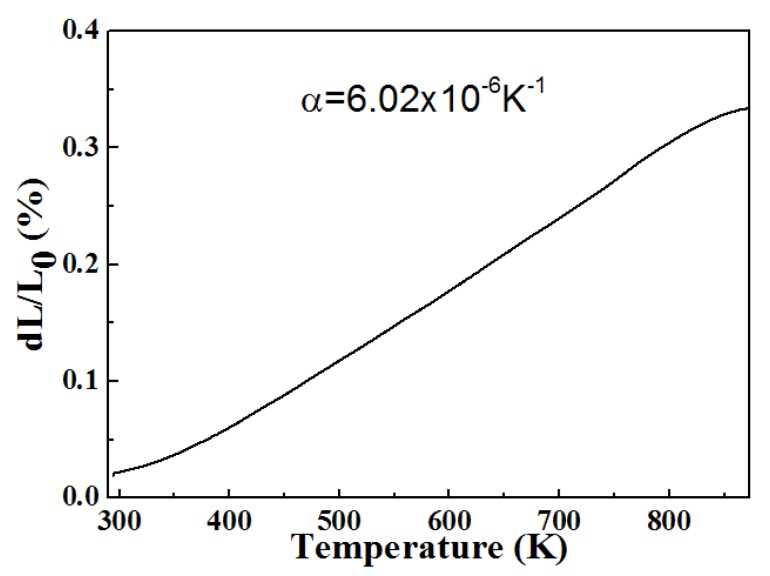
Relative linear length change of YVO_4_ prepared with Y_2_O_3_ and V_2_O_5_ raw materials using the same temperature as Y_2−*x*_Zr*_x_*Mo_3−*x*_V*_x_*O_12_. The linear coefficients of thermal expansions (CTE) is about 6.02 × 10^−6^ K^−1^.

From the SEM images (from *x* = 0.0 to *x* = 0.4), it can be seen that the pores reduced, suggesting that the hygroscopicity decreased with the increased substitution of Zr^4+^/V^5+^ for Y^3+^/Mo^6+^. The low thermal expansion (for *x* = 1.4 and 1.3) could have been related to the formation of two phases of Zr(MoO_4_)_2_ and YVO_4_. Zr(MoO_4_)_2_ present in the NTE [30], while YVO_4_ showed positive thermal expansion (Figure 4), and consequently, they canceled out one another’s thermal expansion and low thermal expansion was obtained.

## 4. Conclusions

The effect substitutions of Zr^4+^/V^5+^ for Y^3+^/Mo^6+^ in Y_2_Mo_3_O_12_ on the hygroscopicity and thermal expansion property were investigated. For the smaller substitution content (Zr*_x_*Y_2−*x*_V*_x_*Mo_2−*x*_O_12_, *x* ≤ 0.5), their crystal structures remained orthorhombic. However, there was crystal water still in lattice. The adsorbed water disappeared with the increase of substitution content (*x* = 0.7). The linear CTEs for *x* = 0.1–0.7 could have been changed linearly with the substitution amount of Zr^4+^/V^5+^ for Y^3+^/Mo^6+^ in Y_2_Mo_3_O_12_. For *x* ≥ 1.0, low thermal expansion, and nonhygroscopicity from RT could be obtained, relating to the two phases of Zr(MoO_4_)_2_ and YVO_4_. The results suggested that bi-substitution could be a better way to reduce the hygroscopicity and to improve the thermal expansion property of Y_2_Mo_3_O_12_.

## Supplementary Materials

The following are available online at https://www.mdpi.com/1996-1944/12/23/3945/s1, Figure S1: XRD patterns of Y_2-*x*_Zr*_x_*Mo_3-*x*_V*_x_*O_12_, for *x* = 0.2, 0.4 and 0.7.

## References

[1] Chen J., Wang F.F., Huang Q.Z., Hu L., Song X.P., Deng J.X., Yu R.B., Xing X.R. (2013). Effectively control negative thermal expansion of single-phase ferroelecrics of PbTiO_3_-(Bi,La)FeO_3_ over a giant range. Sci. Rep..

[2] Yan J., Sun Y., Wen Y.C., Chu L.H., Wu M.M., Huang Q.Z., Wang C., Lynn J.W., Chen Y.L. (2014). Relationship between spin ordering, entropy, and anomalous lattice variation in Mn_3_Sn_1-ε_ Si_ε_C_1-δ_ compounds. Inorg. Chem..

[3] Yao W.J., Jiang X.X., Huang R.J., Li W., Huang C.J., Lin Z.S., Li L.F., Chen C.T. (2014). Area negative thermal expansion in a beryllium borate LiBeBO_3_ with edge sharing tetrahedral. Chem. Commun..

[4] Yuan B.H., Liu X.S., Song W.B., Cheng Y.G., Liang E.J., Chao M.J. (2014). High substitution of Fe^3+^ for Zr^4+^ in ZrV_1.6_P_0.4_O_7_ with small amount of FeV_0.8_P_0.2_O_4_ for low thermal expansion. Phys. Lett. A.

[5] Yan J., Sun Y., Wang C., Chu L.H., Shi Z.X., Deng S.H., Shi K.W., Lu H.Q. (2014). Tensile ductility of nanotwinned austenitic grains in an austenitic steel. Scripta Mater..

[6] Hu L., Chen J., Fan L.L., Ren Y., Rong Y.C., Pan Z., Deng J.X., Yu R.B., Xing X.R. (2014). Zero thermal expansion and ferromagnetism in cubic Sc_1-*x*_M*_x_*F_3_ (M = Ga, Fe) over a wide temperature range. J. Am. Chem. Soc..

[7] Wu M.M., Hu Z.B., Liu Y.T., Chen D.F. (2009). Thermal expansion properties of Ln_2-*x*_Cr*_x_*Mo_3_O_12_ (Ln=Er and Y). Mater. Res. Bull..

[8] Yuan B.H., Liu X.S., Mao Y.C., Wang J.Q., Guo J., Cheng Y.G., Song W.B., Liang E.J., Chao M.J. (2016). Avoiding the intermediate phase Zr_2_WP_2_O_12_ to develop a larger-negative-thermal-expansion-coefficient material Zr_2_W_2_P_2_O_15_. Mater. Chem. Phys..

[9] Ge X.H., Mao Y.C., Liu X.S., Cheng Y.G., Yuan B.H., Liang E.J., Chao M.J. (2016). Negative thermal expansion and broad band photoluminescence in a novel material of ZrScMo_2_VO_12_. Sci. Rep..

[10] Cheng Y.G., Liang Y., Mao Y.C., Ge X.H., Yuan B.H., Guo J., Liang E.J., Chao M.J. (2017). A novel material of HfScW_2_PO_12_ with negative thermal expansion from 140 K to 1469 K and intense blue photoluminescence. Mater. Res. Bull..

[11] Hemberger J., von Nidda H.-A.K., Tsurkan V., Loidl A. (2006). Spin-driven Phonon splitting in bond frustrated ZnCr_2_Se_4_. Phys. Rev. Lett..

[12] Pokharel G., May A.F., Parker D.S., Calder S., Ehlers G., Huq A., Kimber S.A.J., Suriya Arachchige H., Poudel L., McGuire M.A. (2014). Negative thermal expansion and magnetoelastic coupling in the breathing pyrochlore lattice material LiGaCr_4_S_8_. Phys. Rev. B.

[13] Takenaka K., Okamoto Y., Shinoda T., Katayama N., Sakai Y. (2017). Colossal negative thermal expansion in reduced layered ruthenate. Nat. Commun..

[14] Gautam K., Shukla D.K., Francoual S., Bednarcik J., Mardegan J.R.L., Liermann H.P., Sankar R., Chou F.C., Phase D.M., Strempfer J. (2017). Large negative thermal expansion in the cubic phase of CaMn_7_O_12_. Phys. Rev. B.

[15] Song X.Y., Sun Z.H., Huang Q.Z., Rettenmayr M., Liu X.M., Seyring M., Li G.N., Rao G.H., Yin F.X. (2011). Adjustable zero thermal expansion in antiperovskite manganese nitride. Adv. Mater..

[16] Bridges F., Keiber T., Juhas P., Billinge S.J.L., Sutton L., Wilde J., Kowach G.R. (2014). Local vibrations and negative thermal expansion in ZrW_2_O_8_. Phys. Rev. Lett..

[17] Long Y.W., Hayashi N., Saito T., Azuma M., Muranaka S., Shimakawa Y. (2009). Temperature-induced A–B intersite charge transfer in an A-site-ordered LaCu_3_Fe_4_O_12_ perovskite. Nature.

[18] Liu X.S., Yuan B.H., Cheng Y.G., Liang E.J., Zhang W.F. (2019). Combined influences of A^3+^ and Mo^6+^ on monoclinic-orthorhombic phase transition of negative-thermal-expansion A_2_Mo_3_O_12_. J. Alloy. Compd..

[19] Liu X.S., Cheng F.X., Wang J.Q., Song W.B., Yuan B.H., Liang E.J. (2013). Synthesis, thermal expansion and optical properties of (1-*x*) NaAl (MoO_4_)_2_-*x*NaEr(MoO_4_)_2_ ceramics. J. Alloy. Compd..

[20] Yuan B.H., Yuan H.L., Song W.B., Liu X.S., Cheng Y.G., Chao M.J., Liang E.J. (2014). High solubility of hetero-valence ion (Cu^2+^) for reducing phase transition and thermal expansion of ZrV_1.6_P_0.4_O_7_. Chin. Phys. Lett..

[21] Yuan B.H., He X.K., Chen L.L., Wang W.S., Cheng T., Liang E.J. (2018). Electrical properties and dielectric relaxation behavior of zirconium vanadate. Ceram. Int..

[22] Liu X.S., Cheng Y.G., Liang E.J., Chao M.J. (2014). Interaction of crystal water with the building block in Y_2_Mo_3_O_12_ and the effect of Ce^3+^ doping. Phys. Chem. Chem. Phys..

[23] Li Z.Y., Song W.B., Liang E.J. (2011). Structures, Phase Transition, and Crystal Water of Fe_2-*x*_Y*_x_*Mo_3_O_12_. J. Phys. Chem. C.

[24] Liu H.F., Wang X.C., Zhang Z.P., Chen X.B. (2012). Synthesis and thermal expansion properties of Y_2-*x*_La*_x_*Mo_3_O_12_ (*x* = 0, 0.5, 2). Ceram. Int..

[25] Cheng Y.G., Liu X.S., Song W.B., Yuan B.H., Wang X.L., Chao M.J., Liang E.J. (2015). Relationship between hygroscopicity reduction and morphology evolution of Y_2_Mo_3_O_12_ doped with (LiMg) ^3+^. Mater. Res. Bull..

[26] Sahoo P.P., Sumithra S., Madras G., Row T.N.G. (2011). Synthesis, structure, negative thermal expansion, and photocatalytic property of Mo doped ZrV_2_O_7_. Inorg. Chem..

[27] Liu Q.Q., Yang J., Sun X.J., Cheng X.N., Tang H., Li H.H. (2014). Influence of W doped ZrV_2_O_7_ on structure, negative thermal expansion property and photocatalytic performance. Appl. Surf. Sci..

[28] Chen D.X., Yuan B.H., Cheng Y.G., Ge X.H., Jia Y., Liang E.J., Chao M.J. (2016). Phase transition and near-zero thermal expansion in ZrFeMo_2_VO_12_. Phys. Lett. A.

[29] Liu X.S., Yuan B.H., Cheng Y.G., Ge X.H., Liang E.J., Zhang W.F. (2017). Avoiding the invasion of H_2_O into Y2Mo3O12 by coating with C3N4 to improve negative thermal expansion properties. Phys. Chem. Chem. Phys..

[30] Lind C., Wilkinson A.P., Hu Z.B., Short S., Jorgensen J.D. (1998). Synthesis and properties of the negative thermal expansion material cubic ZrMo_2_O_8_. Chem. Mater..

