# Supplementary material for: Substitutions of Zr4+/V5+ for Y3+/Mo6+ in Y2Mo3O12 for Less Hygroscopicity and Low Thermal Expansion Properties
Source: Materials (Basel). 2019 Nov 28;12(23):3945. doi: 10.3390/ma12233945 (PMC6926913; doi:10.3390/ma12233945)
Supplement: Supplementary file 1 [file materials-12-03945-s001.pdf]

materials

Supplementary Materials

Substitutions of Zr^{4+}/V^{5+} for Y^{3+}/Mo^{6+} in $Y_2Mo_3O_{12}$ for Less Hygroscopicity and Low Thermal Expansion Properties

Qiang Ma [1], Lulu Chen [1], Heng Qi [1], Qi Xu [1], Baohe Yuan [1], Xiansheng Liu [2,*] and Lei Xu [1,*]

[1] Collage of Electric Power, North China University of Water Resources and Electric Power, Zhengzhou 450011, China; mqiang1977@ncwu.edu.cn (Q.M.); chenlulu@ncwu.edu.cn (L.C.) hqi1292@163.com (H.Q.); hnxuq@ncwu.edu.cn (Q.X.); yuanbaohe@ncwu.edu.cn (B.Y.)

[2] Henan Key Laboratory of Photovoltaic Materials and Low Dimensional Materials Science Laboratory, Henan University, Kaifeng 475004, China

* Correspondence: liuxiansheng69@126.com (X.L.); xulei@ncwu.edu.cn (L.X.); Tel.: +86-15903782302 (X.L.); +86-13838127525 (L.X.)

Received: 11 October 2019; Accepted: 24 November 2019; Published: date

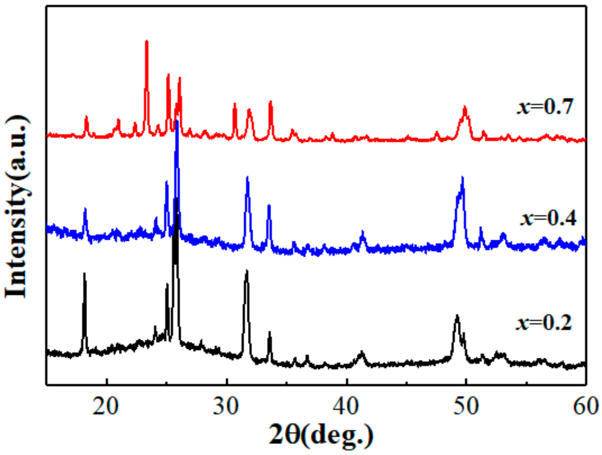

Figure 1. XRD patterns of $Y_{2-x}Zr_xMo_{3-x}V_xO_{12}$, for x = 0.2, 0.4 and 0.7.

The results of other stoichiometric ratios are similar to those mentioned in the submission, for comparison, we only provide some results. For example, the result of XRD for x = 0.2 and 0.4 is similar to x = 0.2 and x = 0.7 is similar to x = 0.5
